# Case report: Combination technique of balloon dilation, membrane excision, and topical mitomycin C for the treatment of nasopharyngeal stenosis in a cat

**DOI:** 10.3389/fvets.2024.1452002

**Published:** 2024-10-02

**Authors:** Ho Hyun Kwak, Sung Min Kim, Lina Yu, Jun Hyung Kim, Heung Myong Woo

**Affiliations:** ^1^Department of Veterinary Surgery, College of Veterinary Medicine, Kangwon National University, Chuncheon, Republic of Korea; ^2^Point Animal Medical Center, Incheon, Republic of Korea

**Keywords:** nasopharyngeal stenosis, balloon dilation, excision, mitomycin C, cat

## Abstract

A two-year-old neutered male Turkish Angora cat presented with respiratory signs, including chronic snoring sounds and dyspnea with open-mouth breathing. Nasopharyngeal stenosis (NPS) was diagnosed based on endoscopy and computed tomography (CT). An attempt was made to break down the membrane, causing stenosis in the nasopharynx through balloon dilation using a valvuloplasty balloon dilation catheter (12 mm × 3 cm) and retroflexed endoscope. The balloon size was selected according to the identified diameter of the stenotic site on nasopharyngeal CT images. The balloon was inflated with radiographic contrast medium and maintained for 2 min; the similar procedure was repeated four additional times. The stenotic membrane was excised after balloon dilation. Topical Mitomycin C (MMC) was then administered to the stenotic region. After 2 weeks, an additional MMC application was repeated to prevent recurrence. The cat remained free of clinical signs without recurrence for 12 months after the most recent procedure. In this study, effective treatment results were obtained using a combination of balloon dilation, membrane excision, and topical MMC for membranous nasopharyngeal stenosis in a cat.

## 1 Introduction

Nasopharyngeal stenosis (NPS) is a relatively uncommon condition in cats, characterized by narrowing or blockage of the nasopharynx and results in breathing difficulties through the nose (1, 2). Its primary cause is scar tissue formation; however, the exact underlying cause is often unknown (3). Chronic inflammatory conditions or congenital malformations associated with the nasopharynx are commonly suspected as the primary causes (1, 4, 5). However, cats often exhibit upper respiratory symptoms at the time of adoption, making it difficult to determine the cause of NPS. Treatments for NPS include surgical resection (4), balloon dilation (1, 3), mucosal advancement flap (6) or placement of an expandable stent across the area (7). However, these can cause various complications including recurrent stenosis, infection, palatal erosion, and oronasal fistulation (8). Scarring after nasopharyngeal surgery can lead to the recurrence of NPS (1). Scar formation results from fibroblast proliferation and excess collagen (9). Mitomycin C (MMC), an antibiotic discovered in 1956, inhibits DNA replication, RNA, and protein synthesis, while also suppressing fibroblast proliferation (10). Due to these properties, it is utilized in human airway surgeries to minimize scar formation (11, 12). In veterinary medicine, a combination of balloon dilation and MMC has been used to treat recurrent nasopharyngeal stenosis in a dog (13). This is the first case report of successful treatment of NPS in a cat by combining topical MMC application, balloon dilation, and surgical excision.

## 2 Case report

A 2-year-old neutered male Turkish Angora cat weighing 4 kg presented with upper airway noise, nasal discharge, and open-mouth breathing. The owners reported that the cat, which was adopted at 4 months of age, had been exhibiting chronic snoring sounds since they first brought him home as a kitten. Despite antibiotic treatment for symptom improvement, the cat was unresponsive to treatment.

Hematological and serum biochemical findings were within the normal ranges. Feline upper respiratory disease real-time PCR performed on nasal exudate samples were positive for Feline Calicivirus and *Mycoplasma Felis*.

**Figure 1:**
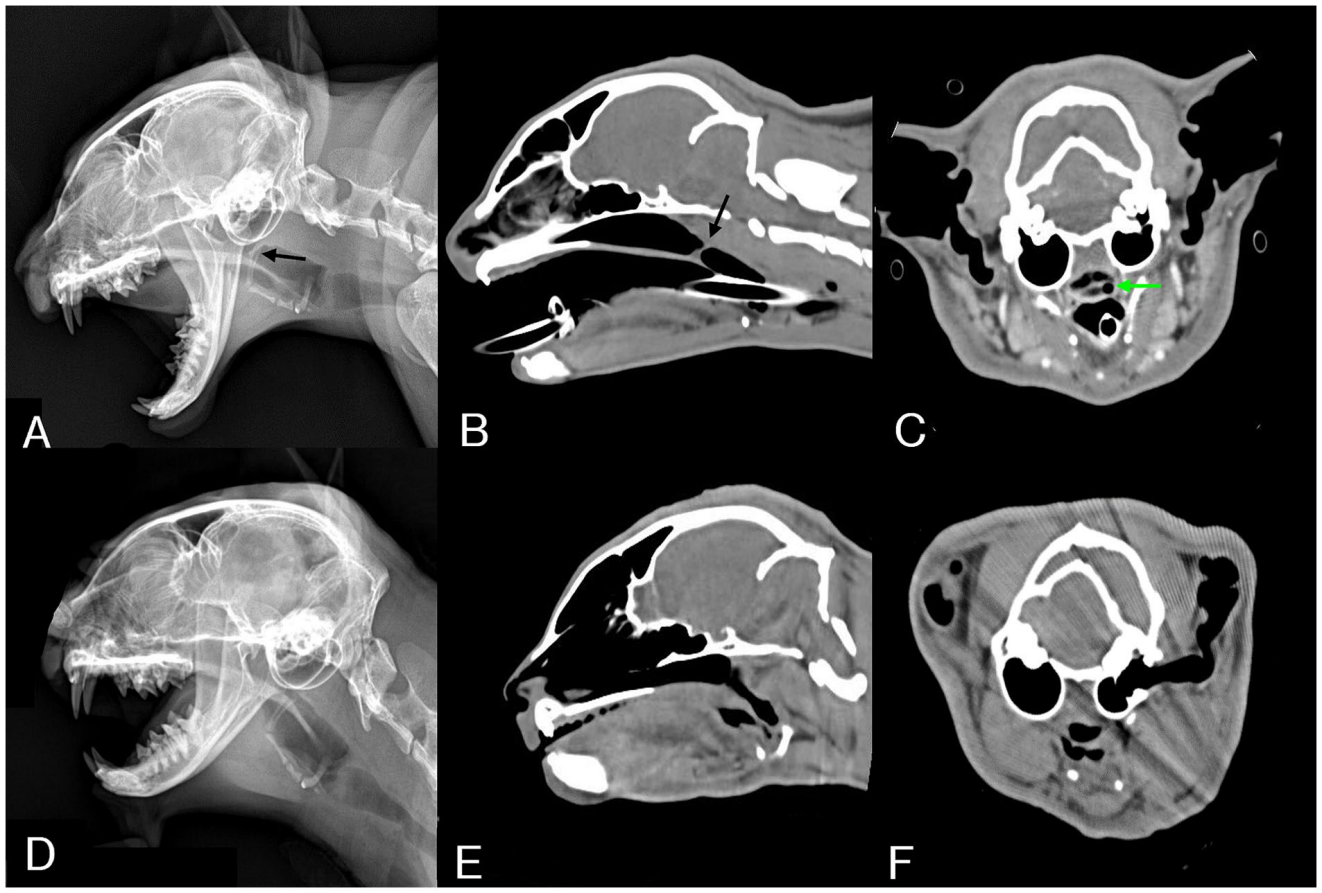
Pre-procedural radiographic and CT images and post-procedural one-month follow-up images. **(A)** On lateral radiography of the head, a thin band-shaped structure (black arrow) is identified in the nasopharynx region. **(B)** Sagittal reconstructed CT image revealing a thin band-like structure (black arrow) crossing the nasopharynx. **(C)** Transverse reconstructed CT image showing a narrow opening (green arrow) of the nasopharynx, measuring 2.5 mm in diameter and deviated to the left from the center. **(D)** On follow-up lateral radiography of the head, the band-like structure that was in the nasopharynx region was not observed. **(E)** Sagittal reconstructed CT image showing patency from the nasopharynx to the pharynx cavity. **(F)** The nasopharyngeal area, which previously showed with stenosis, no longer exhibited any stenosis, and patency was confirmed.

Lateral radiographs of the thoracic cavity, head, and neck were obtained. Radiography revealed a thin band-shaped structure of soft tissue opacity in the nasopharyngeal region, 15 mm rostral to the end of the soft palate (Figure 1A). Additionally, sagittal reconstruction of CT confirmed the presence of a 1 mm thick membranous structure at the dorsal aspect of the soft palate (Figure 1B). Measurements from the computed tomographic images used for treatment planning included a distance of 75 mm from the nasal planum to the stenotic area (Figure 1B), with a width of 11 mm of the nasopharynx in front of the stenotic area, and the narrowest width of the stenotic area (2.5 mm) deviating to the left from the center (Figure 1C). No other anatomical or pathological abnormalities were observed.

**Figure 2:**
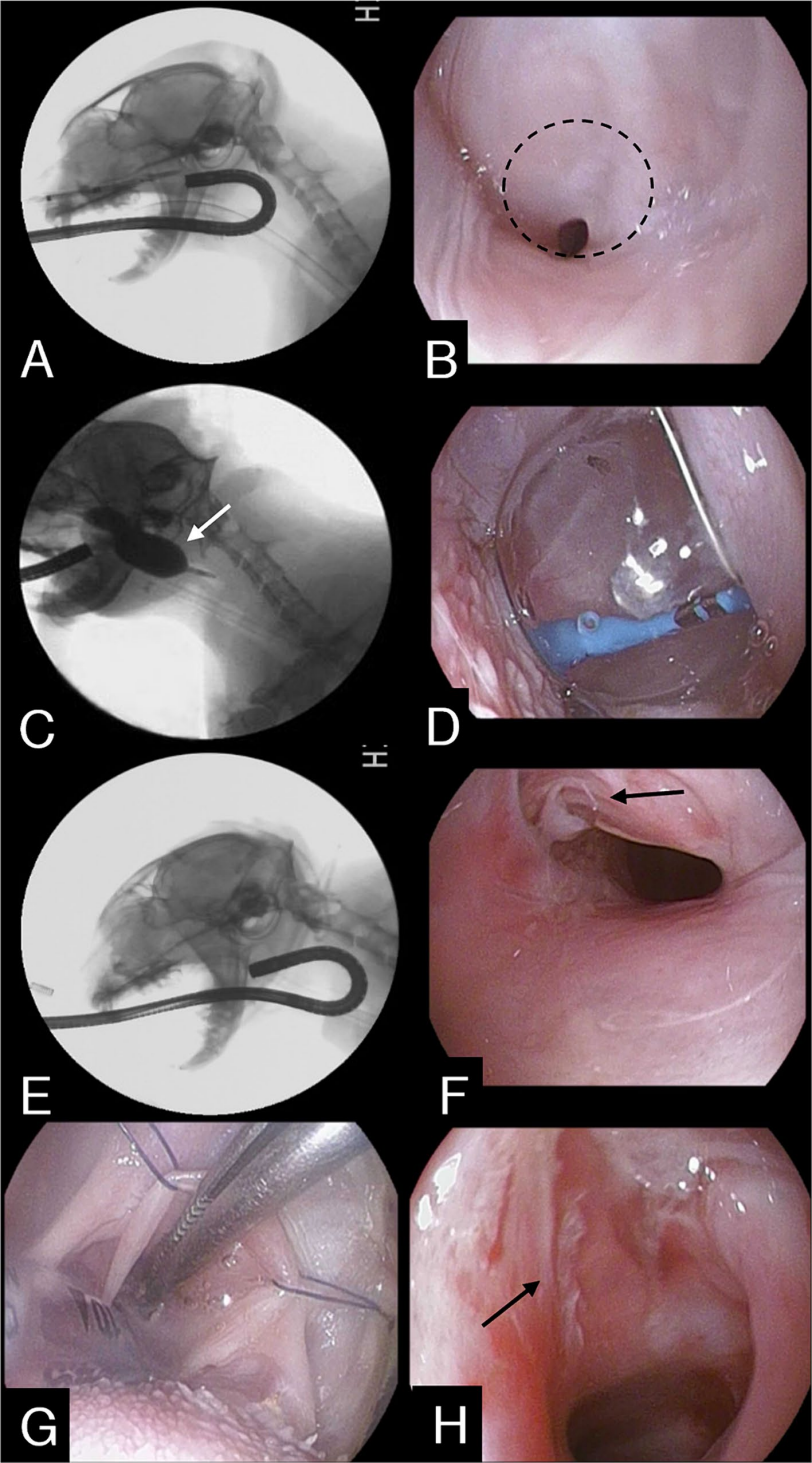
**(A)** Fluoroscopic image during retroflex endoscopy. **(B)** Retroflex endoscopic image of the NPS in the nasopharynx. The dashed line indicates membranous structures constituting nasopharyngeal stenosis. **(C)** The balloon was inflated and maintained (white arrow) for 2 min. **(D)** Same as seen in (C). **(E)** After the procedure, the stenotic area was re-evaluated through an endoscope. **(F)** Endoscopic image showing stenotic lesion being torn apart (black arrow), widening the nasopharyngeal cavity, after completing the entire procedure. **(G)** Stay sutures placed to expose the torn scar tissue. **(H)** During the surgical excision process, partially resected tissue is visible (black arrow).

Nasopharyngeal endoscopy was conducted with the cat in lateral recumbency, using a flexible fiberscope of 5.4 mm external diameter and 1.1 m in length (EG16-K10 Video Gastroscope, Pentax Medical, Tokyo, Japan). Prior to the procedure, the cat was premedicated with butorphanol (0.2 mg/kg, IV), midazolam (0.2 mg/kg, IV) and atropine sulfate (0.02 mg/kg,). Anesthesia was induced using propofol (5 mg/kg, IV) and maintained with 1.2–1.8 percent isoflurane. The endoscope was passed through the mouth into the caudal oropharynx and then reflected dorsally over the soft palate (Figure 2A). Nasopharyngoscopy confirmed a significant accumulation of mucus in the caudal nasopharynx and revealed nasopharyngeal stenosis with a narrow opening in the left corner (Figure 2B).

From these aforementioned findings, the patient was diagnosed with NPS.

A procedure involving balloon dilation was conducted to disrupt the stenosis. Using endoscopic guidance, a guidewire was advanced through the working channel of the endoscope and the stenotic opening under endoscopic visualization with the endoscope in a retroflexed position in the nasopharynx. A straight 0.035-inch guidewire (Weasel wire, Infiniti Medical, Haverford, Pa., United States) was advanced rostrally, where the distal tip was secured at the nostril. A well-lubricated valvuloplasty balloon dilation catheter (PDC507 12 mm X 3 cm BALL CATH TYSHA II STD, B. Braun Interventional Systems Inc., Pennsylvania) was introduced over the guidewire through the nasal opening and passed in an orthograde fashion along the ventral nasal canal to the nasopharynx through the stenotic area until the entire inflatable part was beyond the stenosis. Afterwards, the balloon was inflated with a mixture of 50% iohexol and 50% saline under fluoroscopic guidance, maintained for 2 min, and then deflated (Figures 2C,D). This process was repeated four times to achieve a sufficient airway diameter (Figures 2E,F).

After balloon dilation, the cat was placed in the dorsal recumbent position. Stay sutures were carefully utilized for extracting the soft palate toward the mouth, facilitating access to the nasopharyngeal stenosis (Figure 2G). Torn parts resulting from the balloon procedure were grasped with debakey forceps and retracted, then excised at their attachment points using electrocautery (Figure 2H). Post-procedural examination revealed an enlarged nasopharyngeal passage and mild degree of mucosal hemorrhage in the nasopharyngeal area. Subsequently, 0.4 mg/mL mitomycin C (MMC) (Mitomycin-C Inj., Korea United Pharm, Korea) was applied to the excised tissue area using cotton for 5 min to inhibit tissue regeneration, followed by flushing with saline solution. Recovery from anesthesia was routine and uneventful. To reduce the risk of restenosis from scar formation or secondary infection, the cat was prescribed prednisone (1 mg/kg every 24 h), doxycycline (10 mg/kg every 24 h), and cephalexin (20 mg/kg every 12 h) for two weeks after the procedure.

After two weeks, the cat underwent a second application of MMC in the nasopharynx. The owners reported no respiratory abnormalities after discharge, and the cat’s activity level was normal. The cat was anesthetized using the same protocol as previously described, and an endoscopic examination of the nasopharynx was performed that revealed that the diameter of the stenotic lesion was larger compared to before dilation with mild inflammation. The application of MMC to the affected region was repeated to reduce recurrent scarring, and the cat was discharged without complications.

**Figure 3:**
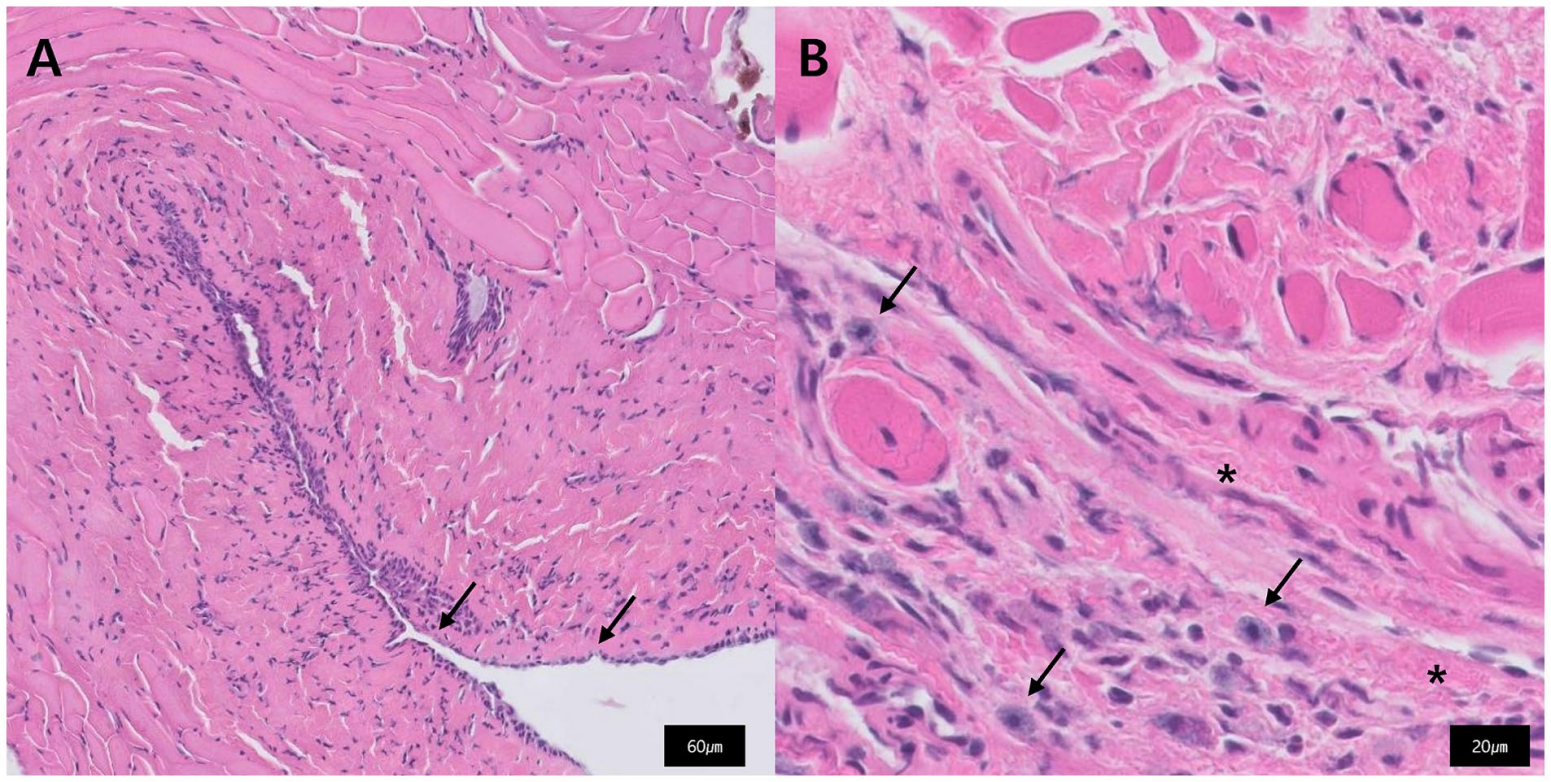
Histopathology of the excised tissue that constituted the nasopharyngeal stenosis. **(A)** Multifocal loss of epithelium is observed (black arrow). **(B)** Fibrous connective tissue (star) and admixed with mast cells (black arrow).

Histopathological examination of the excised tissue revealed multifocal loss of the epithelium (Figure 3A), mild to mild-to-moderate numbers of mast cells and eosinophils admixed with fibrous connective tissue, and edema (Figure 3B). Histological diagnosis indicated chronic inflammatory rhinitis, possibly of allergic origin.

The cat was re-evaluated 4 weeks after the last MMC application. Lateral skull head radiography and CT examination revealed disappearance of the membranous structure (Figures 1D,E). Additionally, patency from the nasopharynx to the oral cavity was confirmed (Figure 1E), with dilation of the narrowed area (Figure 1F).

Twelve months after the last MMC treatment, the owner reported that the cat revealed no symptoms related to stenosis recurrence.

## 3 Discussion

The etiology of nasopharyngeal stenosis (NPS) in cats is not fully understood. However, it can occur as a congenital anomaly, or more commonly, as a secondary inflammatory condition, such as chronic rhinitis, aspiration rhinitis, or a tumor/polyp (8). In cats, infectious agents, including viruses and bacteria, and inflammatory factors can cause nasopharyngeal diseases (14, 15). Severe mucosal ulcerations are often present in virus-related rhinotracheitis and may trigger the development of nasopharyngeal strictures (4, 5, 14). In the present study, the cat exhibited upper respiratory symptoms upon admission. Additionally, the detection of Calicivirus and Mycoplasma in nasal discharge samples suggests that these pathogens may be contributory to the development of NPS.

NPS is diagnosed using retroflex rhinoscopy, with CT providing information regarding the extent of the lesion and aiding in procedural planning (8). Retroflex rhinoscopy has the advantage of directly visualizing the lesion, making its characterization more accurate, while CT scans can evaluate the entire nasal cavity and nasopharynx both rostral and caudal to the stenosis (8, 16). These features complement each other and aid in diagnosis.

Since the first reported surgical correction of NPS, various methods such as balloon dilation (17), metallic stents (5), silicone stents (18), and mucosal advancement (6) have been proposed. Currently, there is no universally accepted treatment, and the choice of procedure may vary depending on the individual patient’s condition.

Regarding the type of NPS, the use of a metallic stent can be effective in cases with a high possibility of recurrence due to an imperforate membrane or extensive stenosis in the nasopharyngeal area. However, the severity of complications, such as oronasal fistula formation, stent fracture or bending, stent migration or removal, and exaggerated swallowing, remains higher (8). Additionally, the location of the lesion can influence the choice of treatment method and the outcome of the procedure (19). In a study involving 46 dogs and cats, it was reported that all patients who experienced complications such as stent bending, and excessive swallowing had lesions located in the caudal one-third of the nasopharynx (1). Furthermore, the success rate of balloon dilation was reported to be lower in cases where the lesions were located in the rostral or middle one-third of the nasopharynx (1).

Surgical treatment of NPS was first introduced in 1988 (4). However, simple resection of stenotic tissue is associated with a high recurrence rate (5, 17, 20). In this study, the NPS lesion was located dorsally to the soft palate and in the caudal third of the nasopharynx, allowing for the excision of torn and ragged tissue using electrocautery following balloon dilation without additional invasive incisions. Surgical excision may be contributory for reducing the residual scar tissue volume while increasing the application of MMC to freshly injured areas (21). Balloon dilation, which is a preliminary step before surgical excision, helped secure an adequate airway diameter and facilitated differentiation between normal nasopharyngeal mucosa and torn membranous tissue constituting the stenosis. In one study, despite attempting various treatment methods including stenting, a canine patient with recurrent stenosis was successfully treated using a combination of balloon dilation and MMC. This study also included the process of removing the remaining adhesion tissue after balloon dilation, which may have enhanced the effectiveness of MMC in removing the stenotic tissue and reducing recurrence (13).

Balloon dilation has been selected as the first treatment option for NPS due to its non-invasive nature and low technical difficulty (16). However, due to the high recurrence rate after a single balloon dilation, multiple dilation procedures or stent placement may be required to achieve long-term resolution of clinical signs (1). In a study of interventional treatments involving 46 dogs and cats with benign NPS or imperforate nasopharynx, balloon dilation was performed in cats without the use of topical MMC, and the success rate, even after multiple procedures, was 50 percent (1).

High recurrence rates associated with both surgical excision and balloon dilation appear to stem from inflammatory responses in damaged tissues, leading to fibroblast proliferation and the formation of scar tissue, which subsequently results in restenosis (22). The scarring response to injury involves fibroblast proliferation and excessive secretion of collagen matrix proteins at the wound site, resulting in fibrosis and clinical formation of a scar (9). Hence, research on NPS has increasingly focused on the modulation of fibrosis, which causes recurrence. Several potential pharmacological modulators have been investigated for their effectiveness in treating NPS. These include systemic antibiotics and steroids, local steroid injections, and MMC administration (13).

MMC is an antitumor antibiotic, and its cellular effects include DNA base pair disruption, inhibition of RNA and protein synthesis, suppression of fibroblast proliferation, and induction of fibroblast apoptosis (10, 23). Clinically, it was first used in pterygium surgery in 1963 and was rediscovered in the 1990s for its utility in ophthalmological procedures in human medicine (10, 24). Subsequently, topical application of MMC following surgery for the treatment of airway stenosis has shown successful outcomes in reducing scar formation (25). However, the intervals or the number of applications for MMC retreatment are not standardized. In a randomized study on laryngotracheal stenosis in humans, it was found that patients who received two applications of MMC had a lower recurrence rate compared to those who received only one application (24). A study using rat models has demonstrated that these suppressive effects last for approximately 2–3 weeks (26). In another human study, the application of MMC at 2-week intervals resulted in successful treatment outcomes in children with refractory esophageal strictures (27). Additionally, a case study reported the successful treatment of dogs with recurrent NPS through one session of balloon dilation followed by MMC treatment (13). We hypothesized that after using balloon dilation to widen the diameter within the nasopharynx, followed by surgical excision to remove stenotic tissue, applying local MMC twice at two-week intervals would lead to reduced scar formation and restenosis.

Cephalexin, a first-generation cephalosporin, is widely used in cats to treat bacterial infections, including feline chronic rhinitis, due to its broad-spectrum activity against bacteria such as Staphylococcus and Streptococcus species (28, 29). However, its therapeutic efficacy can be limited in cases involving Mycoplasma infections, which are potential contributors to secondary bacterial infections (29). Doxycycline, a tetracycline-class antibiotic, is effective against Mycoplasma spp., but less effective against *E. coli* and is only effective against a few Staphylococcus and Streptococcus species (14, 29). In this case, a feline upper respiratory real-time PCR performed on nasal exudate samples revealed positive results for Mycoplasma. To achieve both broad-spectrum antibacterial effects and anti-Mycoplasma activity, a combination of two antibiotics was used as post procedural treatment. Combination therapy for feline chronic rhinitis provides broader coverage but increases the risk of side effects and requires caution due to the rising issue of antibiotic resistance (29, 30). A limitation in this case is that respiratory bacterial cultures were not performed.

To the best of our knowledge, successful treatment of NPS in cats using a combination of balloon dilation, surgical excision, and MMC application has not been reported. In this case, we attempted surgical excision after balloon dilation and administered MMC to reduce scarring, minimize recurrence, and achieve satisfactory outcomes.

For patients with thin membranous stenosis, this combination technique appears to be a minimally invasive and effective treatment method for feline nasopharyngeal stenosis.

## Data Availability

The raw data supporting the conclusions of this article will be made available by the authors, without undue reservation.
